# Effect of various beverages on colour stability with CAD-CAM and light cure provisional restoration - An *in vitro* study

**DOI:** 10.6026/973206300211963

**Published:** 2025-07-31

**Authors:** Saman Shaikh, Anjali Maheshwari, Taku Pugang, Alka Gupta, Harsh Chansoria, Shivani Gupta

**Affiliations:** 1 Department of Prosthodontics and Crown & Bridge, Government College of Dentistry, Indore, Madhya Pradesh, India; 2 Department of Orthodontics and Dentofacial Orthopaedics, Shri Dharmasthala Manjunatheshwara College of Dental Sciences, Dharwad, Karnataka, India

**Keywords:** CAD-CAM, color stability, composite resin, Poly Methyl Methacrylate (PMMA), provisional restoration

## Abstract

The color stability of two provisional restorative materials, Computer-Aided Design-Computer-Aided Manufacturing (CAD-CAM) and Poly Methyl Methacrylate (PMMA) and light-cure composite resins, when exposed to various beverages is od interest. Samples were immersed in tea, coffee, turmeric solution, soft drink and artificial saliva, with color changes measured using a spectrophotometer at 15, 30 and 90 days. The results revealed that CAD-CAM PMMA demonstrated superior color stability, particularly in tea and turmeric, compared to composite material. Thus, the importance of material selection based on aesthetic longevity for provisional restorations is shown.

## Background:

Provisional restorations play a vital role in the overall success of fixed partial denture (FPD) treatment. Between tooth preparation and the final placement of the prosthesis, provisional restorations are given [1]. During this time, it is crucial to maintain the best possible periodontal health, abutment relationships, function and aesthetics. Provisional restorations serve this purpose and thus significantly impact the final outcome of the fixed restorative procedure [2]. In implant prosthodontics, provisional restorations are often required for an extended period of up to six months or longer in certain cases [3]. Additionally, well-designed provisional restorations support guided tissue healing and helps maintain periodontal health, which is particularly critical in areas where high aesthetic outcomes are necessary [4]. Long-term treatment-related discoloration of temporary crowns and bridges can lead to patient dissatisfaction and increased replacement costs [5]. As a result, colour stability is crucial when choosing temporary materials, particularly for aesthetic purposes [6]. A number of variables, such as incomplete polymerization, water sorption, chemical reactivity, the restoration's surface roughness, diet and oral hygiene, can influence the degree of discoloration [7, 8]. The impact of staining agents like tea, coffee and red wine on temporary materials has been the subject of numerous studies. A prior study found that bis-acryl-methacrylate-based resins, a common temporary material, were more colour-stable than methyl/ethyl methacrylate-based resins [9]. As computer-aided design and computer-aided manufacturing (CAD/CAM) technology has advanced various milling systems have been developed [10]. Because polymethyl methacrylate (PMMA) block has better mechanical and physical properties, it has been recommended for use in milling by several studies. This pre-polymerized material promotes good homogeneity without polymerization shrinkage. CAD/CAM PMMA material demonstrated the best colour stability among the various temporary materials, according to a prior study [11]. The question of which kind of material bisacryl composite resins, PMMA, or polyethyl methacrylates has superior colour stability is still up for debate in research. The staining agent's concentration and the length of time the materials are exposed to them both influence the staining intensity. While coffee discolours because the colorants both adsorb and absorb into the restorative material, tea discolours because the polar colorants adsorb onto the surface of the restorative materials. Several studies showed that some resins based on PMMA tend to discolour less than bisacryls and other temporary resins [12]. Therefore, it is of interest to describe the comparative colour stability of various temporary materials, including bisacryl composite resins, PMMA and polyethyl methacrylates, in relation to staining agents and the impact on the longevity and aesthetics of provisional restorations.

## Materials and Methods:

Two provisional restorative materials used in this study are outlined in the table below. The first material, Dentura PMMA Blank, is a CAD-CAM PMMA blank manufactured by Delta India in shade A2. The second material, Te-Econom Plus, is a light-cure composite resin from Ivoclar Vivadent, also in shade A2. Five commonly used beverages and solutions, along with a control of artificial saliva, were chosen to test the staining effects on the materials. These beverages and solutions included tea, coffee, turmeric solution and artificial saliva. A standardized metal mold with a 10 mm diameter and 2 mm thickness was created for the study. For the PMMA samples, a type III dental stone disc model was made by pouring the stone into the mold. After finishing the stone model, it was lab-scanned and then CAD-CAM milled to the desired dimensions. For the light-cure composite provisional material, it was mixed according to the manufacturer's instructions, placed in the mold and cured. After polymerization, the specimens were trimmed and polished for further analysis. The staining solutions used *i.e.* tea, coffee, soft drink and turmeric were prepared in the following concentrations: For preparation of tea solution 2.8 g of tea was added to 150 ml of boiling distilled water. For preparation of coffee solution 2.8 g of coffee was added to 150 ml of boiling distilled water. Soft drink was used as such. For preparation of turmeric solution 0.5 g of turmeric was added to 150 ml of distilled water. In each solution, artificial saliva will be used in order to simulate the oral conditions. 60 samples were prepared and were divided into two groups of 30 samples each (Group A = Cad/ CAN-fabricated PMMA based provisional restorative material and Group B: 30 samples of light cure composite resin based provisional restorative material) To evaluate the color stability in different solutions, 30 specimens of each group is subdivided into five subgroups of 6 specimens each according to the staining solutions used.

## The staining solutions used are:

[1] Subgroup I - A mixture of tea (330 ml) and artificial saliva (660 ml).

[2] Subgroup II - A mixture of coffee (330 ml) and artificial saliva (660 ml).

[3] Subgroup III - A mixture of soft drink (330 ml) and artificial saliva (660 ml).

[4] Subgroup IV - A mixture of turmeric solution (330 ml) and artificial saliva (660 ml).

[5] Subgroup V - Artificial saliva (660 ml).

Specimens will be immersed in their respective solutions at 37°C. The solution was changed every 3 days and stirred twice daily.

The Color stability of the groups was measured pre-immersion, after 15 days, after 1month, after 3 months using a Reflectance Spectrophotometer with CIELAB system. Color difference (ΔE) was calculated from the mean ΔL*, Δa* and Δb* values with the formula:

ΔE = ([L*f - L*i] 2 + [a*f - a*i] 2 + [b*f - b*i] 2)1/2

Where the initial (i) and final (f) are color descriptors and L*, a* and b * are differences in color parameters for the two specimens measured for comparison.

## Statistical analysis:

A General Linear Model (GLM) analysis with repeated measures was conducted to evaluate the effects of time, group and solution on the outcome variable. The experimental design included one within-subjects factor: Time with three levels [ΔE15 (Color difference after 15 days), ΔE30 (Color difference after 30 days), ΔE90 (Color difference after 90 days)] and two between-subjects factors: Group with two levels (COMPOSITE and PMMA) and solution with five levels (Coffee, Soft Drink, Artificial Saliva, Tea and Turmeric). The study employed a fully balanced design with a total of 60 observations, comprising 6 subjects per combination of group and solution. Descriptive statistics were computed to summarize the data across groups, solutions and time points. At ΔE15, the mean score (± standard deviation) for the COMPOSITE group was 9.14 (±2.56), while the PMMA group had a mean of 7.69 (±7.687). At E30_A, the COMPOSITE group demonstrated a higher mean of 10.38(±6.41) compared to 7.27 (±3.59) for the PMMA group. At the final time point, E90_A, the means were more comparable, with COMPOSITE at 7.82(±0.77) and PMMA at 7.90 (±1.92). The complete set of means and standard deviations for each combination of group, solution and time is presented in Table 1 (see PDF).

## Results:

The data presented in Table 1 (see PDF) compares the color stability of composite and PMMA materials when exposed to various beverages over three different time intervals: 15, 30 and 90 days (ΔE15, ΔE30 and ΔE90, respectively). For composite materials, coffee exhibited the highest color difference at 30 days (14.9850), while tea had the highest color difference at 15 days (11.167) and turmeric showed the most significant change at 90 days (8.5600). On average, composite materials showed a total mean color difference of 9.140 for ΔE15, 10.3757 for ΔE30 and 7.824 for ΔE90. For PMMA, tea caused the highest color difference at 15 days (20.150), while coffee showed a smaller change at 15 days (5.633) but a more significant effect at 90 days (8.6417). Overall, PMMA had a mean total color difference of 7.687 for ΔE15, 7.2663 for ΔE30 and 7.9010 for ΔE90. These results suggest varying levels of color stability across both materials and the different beverages used. The analysis of between-subjects effects demonstrated that all main effects and interactions were statistically significant, with very large effect sizes. A significant main effect of Group was observed (F = 721.87, p < .001, partial η^2^ = .935), indicating substantial differences between the COMPOSITE and PMMA groups. Similarly, a significant main effect of Solution was found (F = 1330.48, p < .001, partial η^2^ = .991), reflecting differences across the five solution types. The interaction between Group and Solution was also significant (F = 554.80, p < .001, partial η^2^ = .978), suggesting that the effect of solution varied depending on group membership described in Table 2 (see PDF). The interaction between Group and Time was further examined through estimated marginal means. For the COMPOSITE group, the means at each time point were 9.14 (ΔE15), 10.38 (ΔE30) and 7.82 (ΔE90), indicating an increase from ΔE15 to ΔE30, followed by a decline at ΔE90. In contrast, the PMMA group showed relatively stable means across time points: 7.69 (ΔE15), 7.27 (ΔE30) and 7.90 (ΔE90), suggesting minimal fluctuation over time as shown in Table 3 (see PDF). The findings indicate significant main effects of Time, Group and Solution (all p < .001), highlighting their strong influence on the outcome variable. Importantly, the critical Time x Group x Solution interaction was highly significant (F = 898.33, p < .001, partial η^2^ = .986), demonstrating that the pattern of change over time differed markedly depending on both group and solution combinations. Effect sizes were consistently large across analyses (partial η^2^ > .90), indicating strong practical significance of the observed effects. Tea exhibited a sharp peak at the 15-days mark (ΔE15, blue line), with the highest estimated marginal mean among all solutions and time points, indicating a pronounced immediate effect. This elevated response declined noticeably by 30 days and further by 90 days, suggesting that the effect of tea was strongest initially but diminished over time. Coffee demonstrated relatively low and stable estimated means across all three time points, with only a slight increase observed at 30 days (ΔE30, red line), indicating a minimal time-dependent effect. Soft drink displayed a decreasing trend from 15 days to 30 days, followed by stabilization or a slight increase at 90 days, suggesting an initial drop in effect that levelled off over time. Saliva consistently showed the lowest estimated marginal means across all time points, with very little variation, indicating a negligible or stable effect throughout the observation period. Turmeric presented moderate estimated means that remained fairly stable, with a slight decline from 30 to 90 days, indicating a relatively consistent but mild effect over time. Figure 1 (see PDF) illustrates that the effect of the solutions on the dependent variable varied substantially across time points. The immediate and pronounced effect observed for Tea contrasts with the more stable profiles seen for Coffee and Turmeric. The variations in estimated marginal means across different solutions and time intervals reflect the significant Time x Solution interaction observed in the GLM analysis, confirming that the solutions influence the dependent variable differently over time. As shown in Figure 2 (see PDF), the estimated marginal means for PMMA materials exhibited relatively stable color changes across the time intervals, with minimal fluctuation observed at 15, 30 and 90 days. Similarly, Figure 3 illustrates the color stability of composite materials over the three time intervals, where the largest color difference occurred at 30 days, followed by a decline at 90 days.

## Discussion:

Color stability is a critical aesthetic parameter for provisional restorations, especially in anterior regions or cases where the restoration will remain in place for an extended period. Discoloration not only compromises the esthetics of the restoration but also diminishes patient confidence and satisfaction, often leading to the premature need for replacement or remakes an outcome that is both costly and time-consuming. In the present study, two material categories were selected for evaluation: CAD/CAM-milled provisional materials and light-cured provisional composites. These materials were specifically chosen to reflect modern clinical trends, each addressing distinct advantages in fabrication, polymerization and potential resistance to staining. CAD/CAM-milled materials, typically made from pre-polymerized PMMA blocks, benefit from highly controlled industrial polymerization. This process results in minimal residual monomer, reduced porosity and a more homogeneous internal microstructure, which enhances the material's resistance to fluid uptake and chromogenic staining agents [13]. On the other hand, light-cured provisional composites, such as Revotek and Fusion Flo, offer clinicians precision in handling, rapid chairside setting and high initial esthetics. These materials cure under visible light, often achieving a high degree of conversion, which can result in durable aesthetics. However, their performance under prolonged exposure to staining agents remains a concern. It is well-established that darker-colored restorative materials tend to exhibit better resistance to color changes than lighter shades. Consequently, the specimens in this study were fabricated using the A2 shade to represent a mid-range baseline, which would improve the reliability of color stability assessments. To simulate real-world conditions, the materials were immersed in clinically relevant beverages-coffee, tea, cola/soft drinks, turmeric-mixed with artificial saliva over controlled time intervals. These solutions are widely recognized for their strong chromogenic potential, as they contain high pigment levels, acidity and sugar content. Coffee and tea are frequently identified as the most potent staining solutions, while turmeric and red wine occasionally surpass them in their potential to cause discoloration. Soft drinks, such as cola, have a moderate effect on color stability but may also impact the material surface through acidic erosion. The dual focus on milled PMMA and light-cured composite materials in this study enables a direct comparison between two clinically popular provisional materials that differ significantly in manufacturing techniques, polymerization quality and structural integrity. Milled PMMA is industrially produced, with high-quality polymerization, while light-cured composites are polymerized chairside using visible light, often resulting in more porous, layered structures.

This comparative design provides a robust framework for assessing how each material withstands habitual exposure to staining agents over time, ultimately helping clinicians make more informed material selections for long-term aesthetic performance. In this study, color measurements were conducted using a spectrophotometer to minimize potential error. Spectrophotometers offer detailed spectral reflectance across the visible spectrum, sampling every 10 nm or less, which allows for precise evaluation of color changes. This contrasts with colorimeters, which provide only a broad overview of light absorption at fixed bands. The adoption of the CIE Lab* uniform color space further enhances the study's reliability by providing an isotropic three-dimensional model that reflects perceptual differences and ensures consistency in color change analyses. Additionally, the smoothness and thickness of the specimen surface are known to affect color stability. To standardize these factors, the thickness of provisional restorative materials in this study was maintained at 2 mm. Consistent with prior research, coffee is among the most chromogenic beverages, causing the most noticeable discoloration across different materials. This is due to coffee's high chromogenic potential, which contributes to discoloration through both surface adsorption and absorption. The results of this study align with findings by Coutinho *et al.* [14] who reported that heat-cured PMMA consistently demonstrated superior color stability compared to bis-acryl materials like Protemp 4 and Luxa Crown. In present context, CAD/CAM milled materials, often made from pre-polymerized PMMA blocks, likely benefit from enhanced polymer conversion and reduced porosity, which makes them more resistant to staining agents like tea and soft drinks. The study by Prasad *et al.* [15] further emphasizes the role of polymerization technique and material chemistry in discoloration, showing that materials with a higher degree of conversion and smoother surface finishes exhibit better resistance to staining. Clinical variables, such as oral hygiene and dietary habits, can accelerate discoloration, as seen in the *in vivo* study by Koumjian *et al.* [16] Although this study is *in vitro*, similar principles apply, with beverages containing strong pigments interacting readily with the resin matrix. This interaction is particularly evident in materials with higher water sorption and lower cross-linking density. Lastly, Doray *et al.* (2001) [17] demonstrated that both acrylic and composite provisional materials undergo perceptible color changes under accelerated aging, with some composite-based materials performing worse than acrylics. These findings are highly relevant for long-term temporization scenarios.

## Conclusion:

Both the type of solution and exposure time significantly influences the color stability of provisional restorations. PMMA showed superior resistance to staining compared to composite, especially with prolonged exposure to beverages like tea and turmeric. Thus, the use of CAD/CAM milled PMMA for better color stability in clinical settings is shown.
